# Using health and demographic surveillance for the early detection of cholera outbreaks: analysis of community- and hospital-based data from Matlab, Bangladesh

**DOI:** 10.3402/gha.v9.30834

**Published:** 2016-05-17

**Authors:** Dell D. Saulnier, Lars-Åke Persson, Peter Kim Streatfield, A. S. G. Faruque, Anisur Rahman

**Affiliations:** 1 International Maternal and Child Health, Department of Women's and Children's Health, Uppsala University, Uppsala, Sweden; 2 International Centre for Diarrheal Disease Research, Bangladesh (icddrb), Dhaka, Bangladesh

**Keywords:** syndromic surveillance, early warning, EARS

## Abstract

**Background:**

Cholera outbreaks are a continuing problem in Bangladesh, and the timely detection of an outbreak is important for reducing morbidity and mortality. In Matlab, the ongoing Health and Demographic Surveillance System (HDSS) data records symptoms of diarrhea in children under the age of 5 years at the community level. Cholera surveillance in Matlab currently uses hospital-based data.

**Objective:**

The objective of this study is to determine whether increases in cholera in Matlab can be detected earlier by using HDSS diarrhea symptom data in a syndromic surveillance analysis, when compared to hospital admissions for cholera.

**Methods:**

HDSS diarrhea symptom data and hospital admissions for cholera in children under 5 years of age over a 2-year period were analyzed with the syndromic surveillance statistical program EARS (Early Aberration Reporting System). Dates when significant increases in either symptoms or cholera cases occurred were compared to one another.

**Results:**

The analysis revealed that there were 43 days over 16 months when the cholera cases or diarrhea symptoms increased significantly. There were 8 months when both data sets detected days with significant increases. In 5 of the 8 months, increases in diarrheal symptoms occurred before increases of cholera cases. The increases in symptoms occurred between 1 and 15 days before the increases in cholera cases.

**Conclusions:**

The results suggest that the HDSS survey data may be able to detect an increase in cholera before an increase in hospital admissions is seen. However, there was no direct link between diarrheal symptom increases and cholera cases, and this, as well as other methodological weaknesses, should be taken into consideration.

## Introduction

The World Health Organization estimates that up to 3–5 million cholera cases and between 100,000 and 120,000 cholera deaths occur globally each year, with the highest burden in children under 5 years of age (1–3). In Bangladesh, cholera is endemic and outbreaks are related to the seasonal monsoons and to natural disasters, like flooding (4, 5). In the urban areas of Dhaka and in rural Matlab, at least two epidemics are observed each year, generally between March to May and November to December (6). In Dhaka, both the frequency of outbreaks and the severity of the cases have increased in the last 15 years (7). The incidence of cholera in the population in and around Dhaka is estimated to be between 280 and 474 cases per 100,000 persons (8).

Although much can be done to prevent the transmission of cholera, outbreaks still occur, and timely detection of an outbreak is essential. Earlier detection of an outbreak can lead to reduced morbidity and/or mortality, limit the spread of disease, and lower the cost of an outbreak (9).

Traditional disease surveillance methods rely on clinical diagnoses reported from treatment facilities or providers. Alternatively, syndromic surveillance continuously monitors symptoms or syndromes that act as a proxy for disease through data that are already being collected from a large geographic area and/or population, such as emergency department chief complaints or school absenteeism records (10). Using continuous symptom monitoring as an indication of an outbreak, rather than relying on reported diagnoses, can enable syndromic systems to detect a disease outbreak earlier than more traditional methods of surveillance (9, 10).

Diarrheal disease surveillance in Matlab, Bangladesh, is undertaken by the International Center for Diarrheal Disease Research, Bangladesh (icddr,b), and uses a hospital-based approach (11). However, the Matlab area has also hosted icddr,b's Health and Demographic Surveillance System (HDSS) since 1966, a large-scale health and demographic survey of the local population. The health symptom data continuously collected by the HDSS program could be used in a syndromic surveillance system to detect cholera at the community level, before an increase of patients at the hospital. The objective of this study was to determine whether increases in cholera in Matlab could be detected earlier by using HDSS diarrhea symptom data in a syndromic surveillance analysis, when compared to hospital admissions for cholera.

**Fig. 1:**
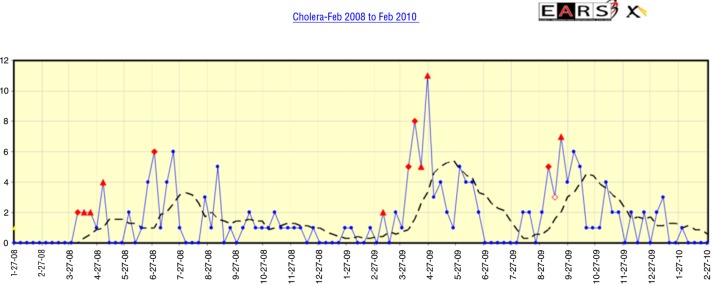
Early Aberration Reporting System analysis of hospital admissions for cholera cases, per week, February 2008 to February 2010. Red symbols: flagged days at differing sensitivities; blue line: number of all cases; black dotted line: average number of cases for the last 7 days; *x*-axis: weeks between February 2008 and February 2010; *y*-axis: number of cholera cases.

## Methods

### Study site and population

This study was an analysis of secondary data. The data came from Matlab, a subdistrict of Chandpur District, Bangladesh. In Matlab, icddr,b has run the HDSS since 1966. The HDSS performs the continuous collection of demographic information – births, deaths, and migrations – on the local population through household surveys (12). In 2013, the population under surveillance by the HDSS was approximately 227,000 people (13).

Since 2008, icddr,b has been collecting information on child morbidity. Ten percent of households with children under 5 years of age are randomly selected by the HDSS program. Community health workers interview mothers in those households and ask if the children have had symptoms of diarrhea in the last 2 weeks. The survey defines *diarrhea* as ‘three or more loose stools per 24 h with or without mucus or blood’ (14).

icddr,b also provides care for diarrheal illnesses to people from and outside the HDSS areas through two hospitals. Matlab Hospital, the main diarrhea hospital located in Matlab Township, provides care to about 20,000–25,000 diarrhea patients each year. Nayergaon Diarrhea Treatment Center, a small diarrhea unit within a subcenter run by icddr,b that is located 15 kilometers away from Matlab Hospital, treats about 3,000 diarrhea patients per year. About 7% of diarrhea cases reported to these two facilities are from the residents of the HDSS area. The remaining cases come from outside the HDSS area.

### Sources of data

This study had two main data sources: HDSS diarrhea symptom data and hospital admissions for diarrhea. HDSS data were collated from the HDSS records for families taking part in the child morbidity component of the system. All complete child morbidity data were used for this study for the period between February 1, 2008, and February 16, 2010. The study considered complete interview forms as those that recorded a positive or negative answer for diarrheal symptoms, the birth date and sex of the child, and the date the survey was completed. The date of survey completion was used as the date of symptom onset in the analysis, because the actual date of symptom onset is not recorded in the surveys. The surveys are collected electronically and uploaded to an online database. They are available immediately after collection.

The hospital admissions data came from the icddr,b Matlab hospital and Nayergaon Diarrhea Treatment Center. The data set included all admissions for diarrhea between February 1, 2008, and February 16, 2010. Standard practice for diarrhea admissions at these hospitals includes routine testing (stool specimen and rectal swab cultures) for selected bacterial pathogens for all admitted patients from the HDSS area and for a systematically selected 2% sample of patients admitted from outside the HDSS area. Also included in the data set were patient's age on date of admission, sex, the outcome of the admission, and the causative pathogen, if tested.

### Statistical analyses

The syndromic analysis was completed using the Early Aberration Reporting System (EARS), version 2.8. The software was developed by the Centers for Disease Control in the United States and was free to download from their website for personal or professional use at the time of this study.

It was beyond the scope of this study to create an algorithm or detect syndromic patterns by hand. An existing algorithm was sought and the EARS software was chosen for its advantages. It uses a quality control statistical method, called *seasonally adjusted cumulative sum*. The calculations are based on the previous 7–10 days as well as the total mean and standard deviation for the time period under study, which allows the program to take into account daily variation as well as seasonality (15). This applies well to the data from Matlab, where cholera and diarrheal illness have strong seasonal patterns. The EARS system also does not require extensive baseline data to work accurately, performing on as little as 7 days of historical data.

Descriptive statistics were done on the HDSS and cholera admissions data. Percentages were calculated, and *p*-values were obtained using Pearson's chi-square tests.

### Ethical considerations

Because this study was a secondary data analysis, the potential ethical implications were limited to those associated with the use of health information; therefore, ethical approval from a review board was not sought or obtained. The continuous data collection for the HDSS is routinely approved by the Ethical Review Committee of icddr,b. The research proposal was accepted by staff at icddr,b, who agreed to share information with the researcher, and by staff at Uppsala University. The data was de-identified before transmission to the researcher, with the minimal amount of information necessary. Access to the data was limited to the project researchers once it was obtained.

## Results

### Hospital admissions

Between February 1, 2008, and February 16, 2010, there were 3,518 admissions for a chief complaint of diarrhea in the two Matlab hospitals (Table 1). The majority of admissions (62%) were in children under the age of 5 years. A causative pathogen was isolated in 6.4% of the admissions. The three detected pathogens were strains of cholera, shigella, and salmonella. Cholera was identified in just over 30% (174 patients) of the admissions tested for a pathogen, with the majority seen during the second year of the study period. Approximately 18% of the cholera cases were seen in children under 5 years of age.

**Table 1:** Counts and percentages for all hospital admissions

	February 2008–January 2009 *n* (%)	February 2009–February 2010 *n* (%)	Both years *n* (%)
Admissions			
Age	2,002 (56.9)	1,516 (43.1)	3,518 (100)
< 1 year	711 (35.5)	433 (28.6)	1,144 (32.5)
1 year	475 (23.7)	272 (18.0)	747 (21.2)
2 years	99 (4.9)	91 (6.0)	190 (5.4)
3 years	39 (1.9)	31 (2.0)	70 (2.0)
4 years	28 (1.4)	18 (1.2)	46 (1.3)
All <5 years	1,352 (67.4)	845 (55.8)	2,197 (62.4)
Sex			
Male	1,067 (53.3)	816 (53.9)	1,883 (53.6)
Female	935 (46.7)	698 (46.1)	1,633 (46.4)
Pathogen			
Cholera	61 (21.2)	113 (43.1)	174 (31.6)
Shigella	183 (63.5)	104 (39.7)	287 (52.2)
Salmonella	44 (15.3)	45 (17.2)	89 (16.2)
Total	288 (100)	262 (100)	550 (100)

* February 1, 2008, to February 16, 2010.

### HDSS surveillance data

There were certain unexpected time periods when no HDSS surveillance data were collected, leaving zero data between the following dates: June 2 to July 24, 2008; September 28 to December 5, 2008; March 9 to May 15, 2009; and July 30 to November 1, 2009.

Despite this, there were 23,579 completed HDSS surveillance forms for children under 5 years, of which 16,280 were identified as unique surveys, where each child was only represented once in the data set. In total, 2,678 surveys had a positive answer for diarrheal symptoms, and 2,493 of those were unique surveys (Tables 2 and 3).

**Table 2:** Counts and percentages for all HDSS surveillance data*

	February 2008–January 2009 *n* (%)	February 2009–February 2010 *n* (%)	Total *n* (%)
Surveys			
Age	14,307 (60.7)	9,272 (39.3)	23,579 (100)
<1 year	2,505 (22.3)	1,518 (30.1)	4,023 (24.7)
1 year	2,219 (19.7)	1,026 (20.3)	3,245 (19.9)
2 years	2,178 (19.4)	878 (17.4)	3,056 (18.8)
3 years	2,200 (19.6)	850 (16.9)	3,050 (18.7)
4 years	2,134 (19.0)	772 (15.3)	2,906 (17.9)
Total unique surveys	11,236 (100)	5,044 (100)	16,280 (100)
Sex			
Male	5,698 (50.7)	2,587 (51.3)	8,284 (50.9)
Female	5,538 (49.3)	2,457 (48.7)	7,995 (49.1)
Total	11,236 (100)	5,044 (100)	16,280 (100)

* February 1, 2008, to February 16, 2010.

**Table 3:** Counts and percentages for all positive HDSS surveillance data*

	February 2008–January 2009 *n* (%)	February 2009–February 2010 *n* (%)	Total *n* (%)
Surveys			
Age	1,751 (65.4)	927 (34.6)	2,678 (100)
< 1 year	422 (26.6)	195 (21.5)	617 (24.7)
1 year	373 (23.5)	243 (26.9)	616 (24.7)
2 years	348 (21.9)	212 (23.4)	560 (22.5)
3 years	261 (16.4)	152 (16.8)	413 (16.6)
4 years	184 (11.6)	103 (11.4)	287 (11.5)
Total unique surveys	1,588 (100)	905 (100)	2,493 (100)
Sex			
Male	814 (51.3)	502 (55.5)	1,316 (52.8)
Female	774 (48.7)	403 (44.5)	1,177 (47.2)
Total	1,588 (100)	905 (100)	2,493 (100)

* February 1, 2008, to February 16, 2010.

### EARS analysis

The cholera admissions data and the HDSS data were entered into the EARS system separately and were analyzed on a per-day basis (Fig. 1).

In order to compare the data sets, the cholera admissions data had to be restricted to the five time periods when the HDSS data were available.

The analysis flagged 43 days when the observed number of cholera cases or positive surveys was greater than the expected count – 23 flagged days in the cholera admissions data set and 20 flagged days in the HDSS data set. Aberrant days were detected in 16 separate months and increases in both data sets were detected in 8 of 16 months (Table 4).

**Table 4:** Dates of days flagged during daily EARS analysis

	HDSS surveys	Hospital admissions
	
	Day of the month	Day of the month
2008		
February	24	
March	11	
April		1
May		9
June		1
August		29, 30
September	20	8, 10, 23
December	14, 17	26
2009		
January	18, 19	29
February	10	25
May	26, 27, 28	30
June		3, 4, 6
November	14, 15, 16, 17	14
December	5, 6, 7, 10	8, 9, 22, 23
2010		
January	11	4, 6
February		2

HDSS, Health and Demographic Surveillance System.

In 5 of the 8 months where flagged days were found in both data sets, the HDSS data flagged days came before the flagged days from the admissions data set. The diarrheal symptom days were flagged from between 1 day and up to 15 days before the flagged admissions days. There were 3 months where the diarrheal symptom days were flagged about 2 weeks earlier than the admission days, and 3 months where the diarrheal symptom days were flagged less than 1 week before the admission days.

## Discussion

The results from the study showed that about half of the days that had an abnormal increase of diarrheal symptoms occurred before days with an abnormal increase of cholera admissions. This finding suggests that diarrheal illness surveys may be able to detect an increase in cholera at the community level before an increase in hospital admissions is seen. When HDSS diarrhea symptom days were flagged before cholera admissions days, the fact that the system detected more than 1 day in a row suggests that the aberration was true (16). HDSS survey data were flagged up to 2 weeks before the cholera admissions days, time that could be used for closer surveillance, preparation, or implementation of control measures.

There were 6 months in the study when flagged days occurred only in the cholera admissions data, with no respective peak in the HDSS data. Explanations for this finding could include that cholera admissions are often adults or children older than 5 years, the rise in admissions being negligible but significant with no impact on the rate of diarrheal symptoms in the survey population, or that there was no appreciable relationship between diarrheal symptoms and hospital admissions.

There were several limitations to this study. First, there was no confirmation that the increases in diarrhea in the community were caused by cholera. Cholera was not always the most common pathogen identified in the hospital admissions, and it is possible that the significant peaks in diarrheal symptoms from the HDSS data were attributable to cases of shigella or salmonella. There is some evidence that shigella is more prevalent in rural areas of Bangladesh, which may partially explain why the proportion of shigella cases was higher than cholera cases (17). HDSS surveillance data may be able to detect outbreaks of other common diarrheal pathogens like shigella, when early detection would still be beneficial.

Second, the small proportion (6.4% in this study) of admissions that were tested for a causative pathogen at the hospitals meant that the majority of admissions had no identified pathogen listed, weakening the ability of the EARS system to detect significant peaks in cholera cases. If more cases of cholera had been identified, it might have been possible to see a stronger relationship between diarrhea peaks and cholera peaks. Furthermore, cholera outbreaks are not officially declared in this setting. Having documented start and stop dates to outbreaks would have strengthened the study by providing a definite method of comparison between these dates and the algorithmically detected peaks.

Third, an unexpected limitation of the study was the long periods of time in the HDSS data where no information was recorded, requiring large portions of both data sets to be cut from the analysis. Having data year-round from the HDSS survey would have contributed to the analysis and results.

Another possible limitation was in the age of the diagnosed cholera cases. On average, only about 20% of the cholera cases were in children under the age of 5 years, when about 60% of all the diarrhea admissions were in the same age group. If the spread of disease in Matlab is skewed towards an older age group, using the diarrheal symptoms of children under 5 years to detect outbreaks may not be an effective method.

Finally, the analysis was completed using the dates that the HDSS surveys were completed, not the date of symptom onset. Since the diarrhea symptoms of the children could have been present up to 2 weeks before the survey completion, the dates flagged in the EARS analysis could be lagging behind the onset of symptoms by 1 to 14 days. A more accurate method would be to use the date of symptom onset, if it were available.

Larger questions arose during this study. Although it would be possible to start a syndromic surveillance program in this setting, Matlab is already known for its low case fatality rate and good management of cholera outbreaks. For example, the cause of death data collected by the HDSS showed that no children under 5 years of age died due to diarrhea in 2014 (13). Earlier detection of cholera increases may have a limited benefit in this setting. In many other settings, especially low-income ones, there are no existing sources of data that could be used for a syndromic surveillance system without a massive input of time and resources in planning, structuring, and running a new program. It is questionable whether it would be beneficial to formulate a syndromic system when the resources needed to develop a system like this might be put to better use in another area, such as prevention or treatment.

To the researchers’ knowledge, this is the first study to look at the use of a syndromic surveillance system for cholera and in Bangladesh. It is also one of a smaller number of studies that have used non-clinical data as a source for syndromic surveillance, and further one where self-reported symptom data has been used. It is therefore difficult to draw concrete conclusions or comparisons with other similar studies. However, this study shows that using community-based symptom data could be beneficial to the earlier detection of increases in cholera and, despite its lack of strong results, may contribute to the overall knowledge in this area.

## Conclusions

Using the HDSS survey data from Matlab in a syndromic surveillance analysis showed that it was possible to identify significant increases in diarrhea and that about half of these diarrheal peaks were occurring up to 2 weeks before peaks in cholera cases, suggesting that HDSS survey data may be able to contribute to the early detection of cholera cases. However, the limitations of this study are considerable. Further research would need to show a clear link between the rises in diarrheal symptoms and cholera cases and would need to correct the methodological and data-based weaknesses before any firm conclusions about the timeliness of HDSS data source could be drawn.
